# Short-term mechanical support with the Impella 5.x for mitral valve surgery in advanced heart failure—protected cardiac surgery

**DOI:** 10.3389/fcvm.2023.1229336

**Published:** 2023-07-11

**Authors:** Anja Osswald, Sharaf-Eldin Shehada, Alina Zubarevich, Markus Kamler, Matthias Thielmann, Wiebke Sommer, Alexander Weymann, Arjang Ruhparwar, Mohamed El Gabry, Bastian Schmack

**Affiliations:** ^1^ Department of Thoracic and Cardiovascular Surgery, West-German Heart and Vascular Center Essen, University Duisburg-Essen, Essen, Germany; ^2^ Department of Cardiac Surgery, University Hospital Heidelberg, Heidelberg, Germany; ^3^ Department of Cardiothoracic, Transplant and Vascular Surgery, Hannover Medical School, Hannover, Germany

**Keywords:** Impella, short-term mechanical circulatory support, mitral valve surgery, high-risk surgery, post-operative low cardiac output, advanced heart failure, protected cardiac surgery

## Abstract

**Introduction:**

Surgical treatment of patients with mitral valve regurgitation and advanced heart failure remains challenging. In order to avoid peri-operative low cardiac output, Impella 5.0 or 5.5 (5.x), implanted electively in a one-stage procedure, may serve as a peri-operative short-term mechanical circulatory support system (st-MCS) in patients undergoing mitral valve surgery.

**Methods:**

Between July 2017 and April 2022, 11 consecutive patients underwent high-risk mitral valve surgery for mitral regurgitation supported with an Impella 5.x system (Abiomed, Inc. Danvers, MA). All patients were discussed in the heart team and were either not eligible for transcatheter edge-to-edge repair (TEER) or surgery was considered favorable. In all cases, the indication for Impella 5.x implantation was made during the preoperative planning phase.

**Results:**

The mean age at the time of surgery was 61.6 ± 7.7 years. All patients presented with mitral regurgitation due to either ischemic (*n* = 5) or dilatative (*n* = 6) cardiomyopathy with a mean ejection fraction of 21 ± 4% (EuroScore II 6.1 ± 2.5). Uneventful mitral valve repair (*n* = 8) or replacement (*n* = 3) was performed via median sternotomy (*n* = 8) or right lateral mini thoracotomy (*n* = 3). In six patients, concomitant procedures, either tricuspid valve repair, aortic valve replacement or CABG were necessary. The mean duration on Impella support was 8 ± 5 days. All, but one patient, were successfully weaned from st-MCS, with no Impella-related complications. 30-day survival was 90.9%.

**Conclusion:**

Protected cardiac surgery with st-MCS using the Impella 5.x is safe and feasible when applied in high-risk mitral valve surgery without st-MCS-related complications, resulting in excellent outcomes. This strategy might offer an alternative and comprehensive approach for the treatment of patients with mitral regurgitation in advanced heart failure, deemed ineligible for TEER or with need of concomitant surgery.

## Introduction

Mitral valve regurgitation (MR) occurs in approximately one third of patients with chronic heart failure (1). Moreover, MR can contribute to the progression of heart failure. Especially in patients with heart failure with a reduced ejection fraction (HFrEF), MR is a predictor for high morbidity and mortality (2). Both pathologies cause a volume overload of the LV with subsequent disease progression.

MR in combination with symptomatic heart failure is initially treated with medical and cardiac resynchronization therapy. In case of progression, despite best medical treatment, either transcatheter edge-to-edge mitral valve repair (TEER) or surgery becomes indicated. Mitral valve surgery in advanced heart failure remains challenging since patients are at great risk of developing a perioperative low cardiac output syndrome (LCOS) or even postcardiotomy cardiogenic shock (PCCS), which is associated with a significant further increase in morbidity and mortality (3).

The Impella 5.0 and 5.5 (5.x) are microaxial, minimally invasive short-term mechanical circulatory support systems (st-MCS) that provide up to 5.5 L/min blood flow (4). The Impella 5.x is designed for partial or full flow mechanical support in left ventricular failure by creating a transaortic unloading of the left ventricle (LV). Major advantages as compared to other st-MCS are the significant reduction in cardiac workload and myocardial oxygen consumption, improving cardiac recovery as well as the ability of full mobilization (5). Implantation can be performed through the axillary artery or directly into the aorta (only Impella 5.5). With the possibility of up to 30 days of treatment, the Impella provides an excellent bridge-to-recovery device without the serious disadvantages of postoperative patient immobilization or the need for extracorporeal circulation (6).

To reduce postoperative LCOS in patients with MR and advanced heart failure, the initial approach of mitral valve surgery with pre-emptive Impella 5.x was established at our institution (7). Moreover, this concept might allow for further enrolment of patients towards surgical therapy of complex structural heart disease with improved outcomes and lasting results. The aim of this study was to analyze the outcome and adverse events of the first series of patients who underwent high risk mitral valve repair or replacement supported by a surgically implanted Impella device.

## Methods

### Study design and population

Between July 2017 and April 2022, 11 consecutive patients underwent high-risk mitral valve surgery for MR supported with an Impella 5.0 or 5.5 ® system (Abiomed, Inc. Danvers, MA). All patients suffered from severe MR (Vena contracta ≥0.7 cm, effective regurgitant orifice area ≥40 mm^2^, regurgitant volume ≥60 ml) and advanced heart failure, with an LVEF ≤25% in preoperative transesophageal echocardiography and NYHA (New York Heart Association) functional class III-VI despite optimal medical treatment (8). All patients were discussed in the multidisciplinary heart team prior to surgery and were either not eligible for transcatheter edge-to-edge repair-procedure or surgery was favored and/or a concomitant procedure was indicated. In all cases, decision for concomitant Impella 5.x implantation was made pre-emptively in the preoperative planning stage to prevent perioperative LCOS. To evaluate the concept of pre-emptively Impella support in patients with high-risk MV surgery, all patients who received an Impella device after the initial surgery due to hemodynamic instability on the intensive care unit were not included in this observational, retrospective study. Patient demographics, clinical characteristics, intraoperative and postoperative hemodynamic parameters and outcomes, adverse events, mortality, as well as echocardiographic follow-up data were evaluated.

The study was approved by institutional ethics board of the University Duisburg-Essen (NO.: 22-10526-BO) and the University Hospital of Heidelberg (S-759/2021) and individual consent for this retrospective analysis was waived.

### Operative technique

At start of surgery, before CPB, a 10 mm vascular graft (Vascutek Terumo, Renfrewshire, Scotland, UK) was anastomosed to the right axillary artery with 6/0 prolene running suture in an end-to-side fashion. Depending on the planned surgical procedure, access to the heart was gained via a median sternotomy or right lateral mini thoracotomy. Access to the mitral valve was established via a transatrial approach in cases of isolated MV surgery or via a transseptal approach if concomitant tricuspid valve surgery was necessary. All surgeries were performed on cardiopulmonary bypass (CPB), myocardial protection was achieved with cold crystalloid cardioplegia. Cannulation in cases with sternotomy was performed in a standardized fashion via the ascending aorta and right atrium. For minimally invasive surgery, the femoral artery and vein were used as the cannulation site. Impella 5.0 or 5.5 was then implanted via the right axillary artery (10/11), as previously described or directly into the aorta (only Impella 5.5) (9). In short, during reperfusion and after description of a sufficient mitral valve surgery result, the Impella device was implanted via the 10 mm graft. Insertion over a guidewire and positioning of the Impella pump in the left ventricle were performed under transesophageal echocardiography- and fluoroscopy guidance. The vascular graft was then shortened to the skin level, and the system was secured appropriately. In case of direct aortic implantation (1/11, Impella 5.5), the 10 mm vascular graft was sewn end-to-side to the distal ascending aorta, at least 7 cm above the aortic valve and subsequently tunneled to the right neck, supraclavicularly. With this implantation technique, the aortic cannula must be placed distally to allow for sufficient space to cross-clamp the aorta. Impella was started with a low-level support. CPB support was gradually decreased while Impella flow simultaneously increased. After weaning off CBP, heparin was partially reversed, aiming for the recommended activated clotting time of 160–180 s.

### Weaning strategy of Impella 5.x

**Figure 1:**
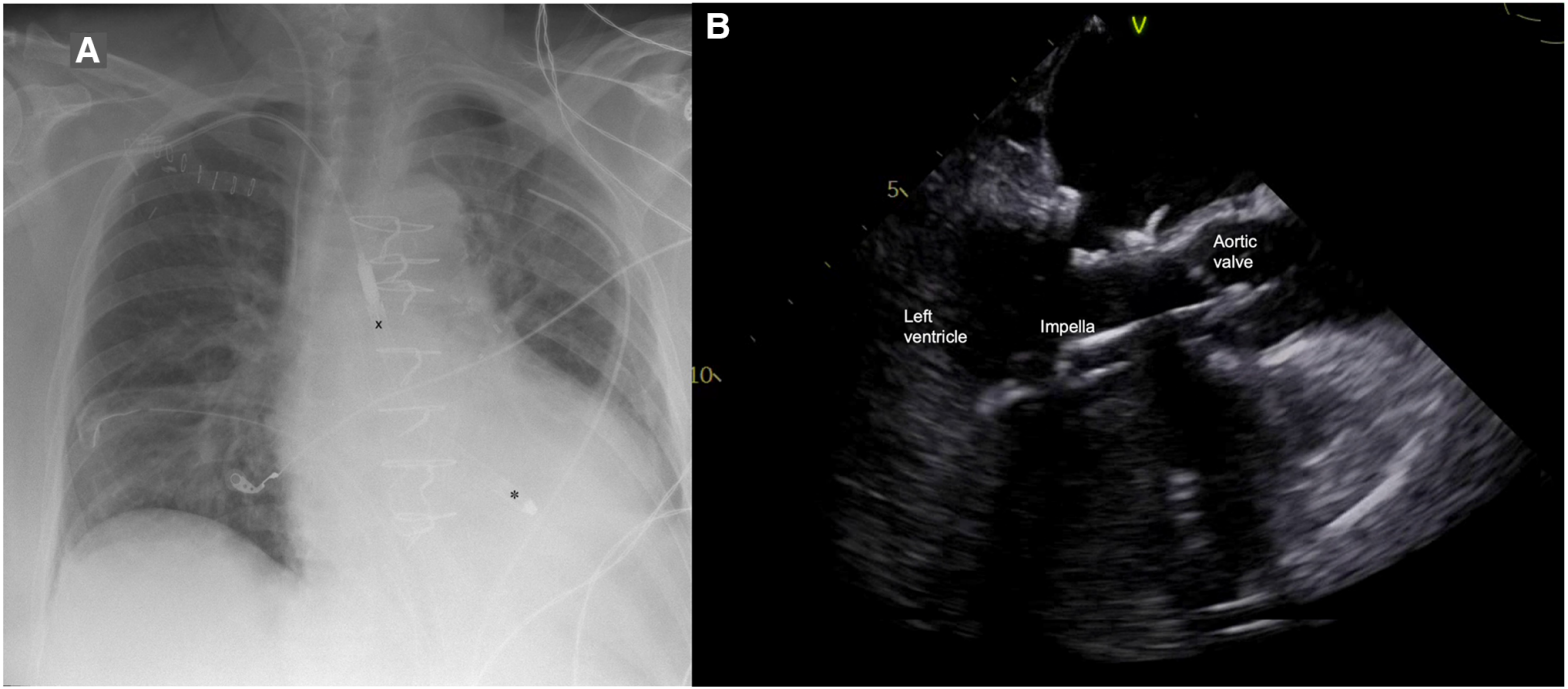
(**A**) Postoperative chest x-ray with Impella 5.5 pump (X outflow, ✽ inflow); (**B**) Impella position in transesophageal echocardiography.

After surgery, patients were stabilized on Impella support with the aim to first reduce catecholamines to avoid side effects, and secondly to reduce direct hemodynamic support level. Patients were evaluated on a daily basis if weaning of Impella support was possible. Hemodynamic and clinical parameters used to guide weaning included: cardiac index >2 L/min, mean arterial pressure >65 mmHg, central venous oxygen saturation >65%, sufficient urinary output >0.5 ml/kg/hr, adequate capillary refill time, serum lactate <20 mg/dl and laboratory results that reflect organ function: creatinine, liver enzymes. Impella position, adequate unloading of the LV, and LVEF were assessed echocardiographically. When hemodynamic stability was achieved, weaning was started in a stepwise fashion by reducing the performance levels, only 1–2 levels at each time. After reduction of Impella support, evaluation of the left ventricular function by echocardiography, as well as hemodynamic measurements (Figure 1). Weaning was performed gradually until performance level P2. The patient had to be stable for 24 h on level P2, before removing the pump either bedside in the intensive care unit or in the operating room under local anesthesia.

### Statistical analysis

Statistical analysis was performed using SPSS (version 27, SPSS, Chicago, IL). Continuous variables are presented as means ± standard deviations. Categorical variables are presented as absolute numbers and percentages. The normality of distributions was checked with the Kolmogorov–Smirnov Test. A paired sample *t*-test was used to compare the pre- and postoperative values. A *p-*value <0.05 was considered statistically significant.

## Results

### Baseline characteristics and preoperative data

Preoperative patient characteristics are displayed in Table 1. A total of 11 patients (45.5% female, 54.5% male) underwent mitral valve surgery with preventive Impella implantation. Mean age at the time of surgery was 61.6 ± 7.7 years. All patients suffered from severe MR and advanced heart failure with a mean left ventricular ejection fraction (LVEF) of 21 ± 4% due to either dilatative (54.5%) or ischemic (45.5%) cardiomyopathy. All patients had multiple comorbidities, resulting in a mean EuroScore II of 6.1 ± 2.5. On admission all patients were in NYHA functional class III or IV, with an INTERMACS class of IV (2/11), V (7/11) or VI (2/11).

**Table 1:** Preoperative patient characteristics.

Demographics	
Age (y)	61.6 ± 7.7
Male	6 (54.5%)
BMI	26.4 ± 5.7
Characteristics	
Underlying cardiomyopathy	
Dilative	6 (54.5%)
Ischemic	5 (45.5%)
Dyspnea	
NYHA III	5 (45.5%)
NYHA IV	6 (54.5%)
Pulmonary hypertension	9 (81.8%)
Cardiovascular risk factors	
Arterial Hypertension	11 (100%)
Diabetes mellitus	2 (18.2%)
Coronary artery disease	6 (54.5%)
Atrial fibrillation	3 (27.3%)
Previous sternotomy	0 (0%)
Echocardiographic findings	
LVEF (%)	21 ± 4
Aortic regurgitation	1 (9.1%)
Mitral regurgitation	
III	6 (54.5%)
IV	5 (45.5%)
Mitral stenosis	0 (0%)
Tricuspid regurgitation	
I–II	7 (63.6%)
III–IV	3 (27.3%)
EuroScore II	6.1 ± 2.5
STS-Score	5.2 ± 4.2

BMI, body mass index; LVEF, left ventricular ejection fraction; NYHA, New York Heart Association functional class; STS-Score, society of thoracic surgeons score.

### Operative details

Surgery was performed in an elective (63.6%) or urgent (36.4%) setting (Table 2). Mitral valve repair was feasible in 72.7% of the cases. Isolated mitral valve repair was performed in 5/11 (45.5%) patients, three of whom via right anterolateral thoracotomy. All other patients (6/11) required concomitant surgery, either coronary artery bypass grafting, aortic valve replacement, or tricuspid valve repair.

**Table 2:** Operative data.

Operative data	
Urgency of procedure	
Elective	7 (63.6%)
Urgent	4 (36.4%)
Emergency	0 (0%)
Mitral valve procedure	
Repair	8 (72.7%)
Ring	4 (36.4%)
Cosgrove band	4 (36.4%)
Replacement	3 (27.3%)
Impella pump	
5.0	5 (45.5%)
5.5	6 (54.5%)
Concomitant procedure	
Tricuspid valve repair	2 (18.2%)
Aortic valve replacement	1 (9.1%)
CABG	4 (36.4%)
Surgical access	
Median sternotomy	8 (72.7%)
Right anterolateral thoracotomy	3 (27.3%)
CBP time (min)	159 ± 55
Aortic cross-clamp time (min)	73 ± 30

CABG, coronary artery bypass grafting; CBP, cardiopulmonary bypass.

### Impella support

**Figure 2:**
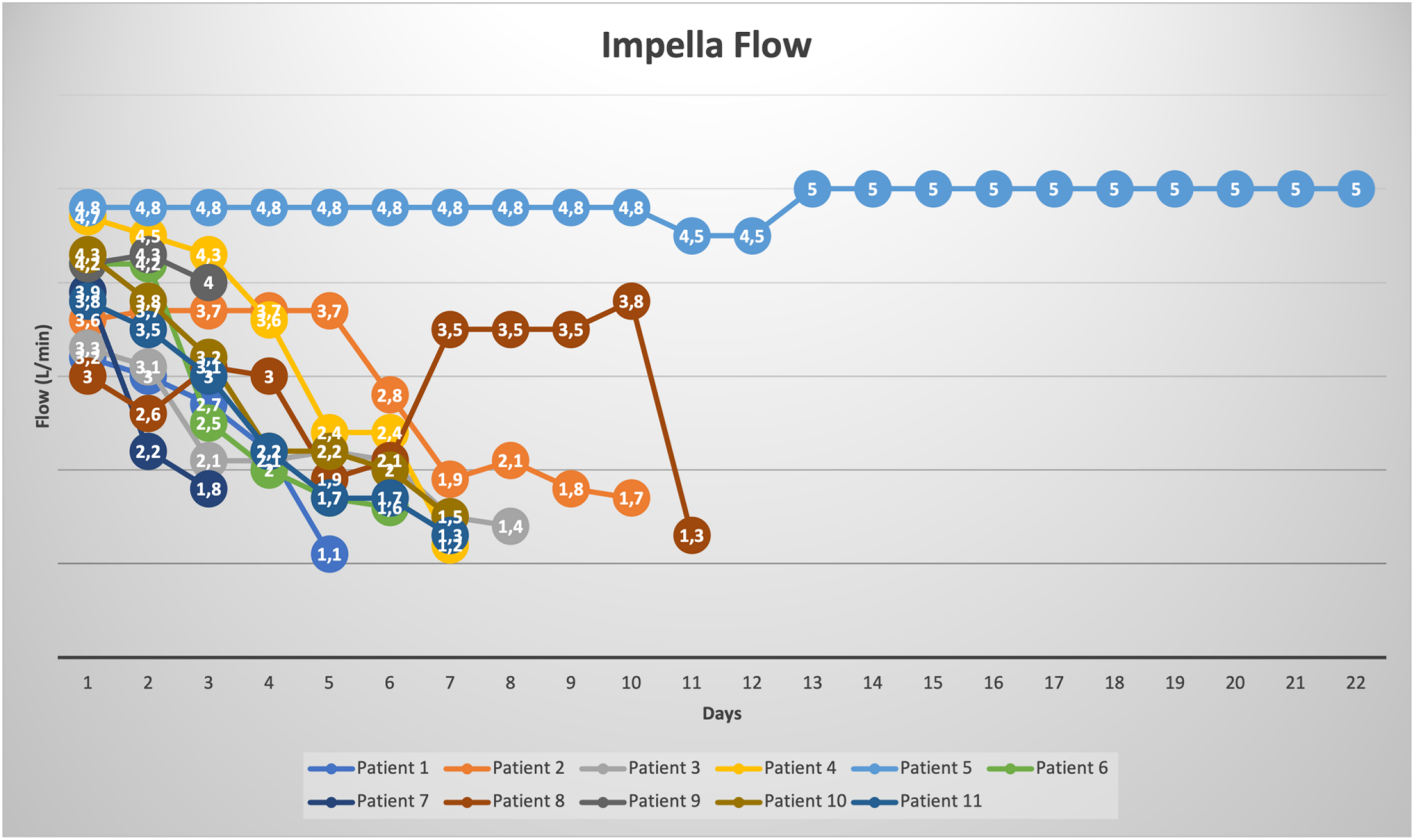
Impella flow rates (L/min) for each patient during the time of st-MCS.

The Impella related data is demonstrated in Table 3. Mean duration of Impella support was 8 ± 5 days (Figure 2). 10/11 patients were weaned from Impella support. In one patient, with preoperative LVEF of 5%–10%, Impella weaning was not achieved. No device related complications occurred, in particular no cerebrovascular accident, vascular complications, no aortic valve injury and no thrombosis, exchange or dislocation of the pump. All, but one patient, were mobilized to different degrees of physical therapy while on Impella support. All patients received initial catecholaminergic support. Four patients (36.4%) were treated with a calcium sensitizer (Levosimendan®) over 24 h prior to weaning.

**Table 3:** Impella related data, LVAD: left ventricular assist device.

Impella Characteristics	
Type of Impella support	
5.0	5 (45.5%)
5.5	6 (54.5%)
Duration of support (days)	8 ± 5
Impella related complications	0 (0%)
Inotropic support	
Noradrenaline	11 (100%)
Dobutamine	10 (90.9%)
Epinephrine	6 (54.5%)
Milrinone	3 (27.3%)
Survival to Impella explantation	10 (90.9%)
Bridge-to-recovery	10 (90.9%)
Bridge-to-LVAD	0 (0%)

### Postoperative outcome

Postoperative outcomes and adverse events are displayed in Table 4. Mean ICU stay was 10 ± 5 days. No postoperative myocardial infarction or stroke was observed in our cohort. Mean preoperative creatinine was 1.16 ± 0.69 mg/dl and slightly increased postoperatively to a maximum of 1.66 ± 1.41 mg/dl (*p*-value = 0.249). Acute kidney injury requiring temporary dialysis occurred in 3/11 patients. Preoperative lactate was 5.95 ± 5.64 mg/dl compared to 9.39 ± 7.54 mg/dl 24 h postoperatively (*p*-value = 0.021). Two patients had to be reintubated but were successfully weaned from the respirator thereafter. Two wound infections occurred, one in the groin after femoral artery cannulation, and one on the leg at the saphenectomy site. Both wound infections were treated with antibiotics, no surgical revision was necessary.

**Table 4:** Postoperative outcomes and adverse events.

Postoperative Outcomes	
Bleeding—rethoracotomy	4 (36.4%)
Impella-related	0 (0%)
Atrial fibrillation	5 (45.5%)
Ventricular fibrillation	2 (18.2%)
Pacemaker implantation	0 (0%)
Stroke	0 (0%)
Myocardial infarction	0 (0%)
Respiratory insufficiency	
Re-intubation	2 (18.2%)
Pneumonia	2 (18.2%)
Renal failure	3 (27.3%)
Conservative treatment	0 (0%)
Hemofiltration	3 (27.3%)
Infections	
Sepsis	1 (9.1%)
Wound infection	2 (18.2%)
ECMO support	1 (9.1%)
ICU stay (days)	10 ± 5
In-hospital death	1 (9.1%)
LVEF in follow up (%)	33 ± 5

ECMO, extracorporeal membrane oxygenation; ICU, intensive care unit; LVEF, left ventricular ejection fraction.

Survival to discharge was 90.9% (10/11). In one patient veno-arterial extracorporeal membrane oxygenation (VA-ECMO) support became necessary due to bleeding from the left main bronchus and consecutive respiratory failure on postoperative day 9 while on Impella support. In this case neither Impella nor VA-ECMO weaning was achieved, and the patient died of multiorgan failure on postoperative day 22.

Echocardiographic follow up was available in 8/11 patients after a median of 3.7 months (interquartile range: 1.1; 10.7). In these patients LVEF significantly improved from 23 ± 3% preoperatively to 33 ± 5% in the follow up (*p*-value <0.001).

## Discussion

In this retrospective study, we present our experience with the preventive use of the Impella 5.x device in patients with advanced heart failure undergoing high-risk mitral valve surgery in order to achieve “protected cardiac surgery”. The underlying concept was a bridge-to-recovery therapy with the Impella device to prevent perioperative LCOS and to allow early LV remodeling after MV surgery.

One of the main determinants of eligibility for heart surgery is LVEF. In severe MR, LVEF is mostly overestimated due to the increase of backwards ejection flow while forward EF is reduced, resulting in malperfusion. Moreover, this deficit in evaluation increases with the severity of MR (10). Therefore, LVEF may not be an adequate indicator to assess eligibility for surgery in patients with HFrEF and severe MR, although it is still frequently used as a decisional indicator.

Treatment of MR and advanced heart failure remains challenging, but studies have shown superiority of interventional treatment compared to medical treatment alone (11, 12). In high-risk patients with HFrEF, TEER has evolved to an alternative to surgical treatment. Results are promising, but the mid- and long-term freedom from recurrence of MR is not favorable. In cases of concomitant surgery or when TEER is not feasible or unlikely to achieve a good result, surgery becomes inevitable. Therefore, guidelines recommend that patients with advanced heart failure and MR be evaluated in a multidisciplinary heart-team (13). In our study eligibility for TEER was discussed for all patients, but heart-team opted for a surgical approach.

Despite high-risk constellation, all but one patient, could be weaned off Impella support, no bridge to a durable assist device became necessary and survival to discharge was 91%. Given the advanced stages of heart failure, these results are very promising. Reported survival rates in Impella studies with heart failure vary widely, depending on several factors, such as underlying diagnosis, acute or chronic heart failure, individual patient risk and timing of implantation (6, 14–16). An evaluation of the first 200 patients treated with the Impella 5.5 in the US showed improved outcomes and survival rates, especially in patients with PCCS (17). Based on the high likelihood of PCCS in our patient cohort, the decision for Impella implantation was made preoperatively. The underlying concept was to provide hemodynamic support in the peri- and postoperative phase, and thereby reduce the risk of LCOS and its complications.

Possible treatment options of LCOS and PCCS are pharmacological support with inotropes and vasopressors and/or st-MCS with VA-ECMO or Impella. Despite the necessity of these treatments, all are associated with certain risks and complications.

Common side effects of catecholamines include excessive vasoconstriction with malperfusion of the extremities, mesenteric organs or kidneys, dysrhythmias, myocardial ischemia, increased oxygen demand and hyperglycemia. Complications of VA-ECMO therapy include bleeding, thromboembolism, SIRS, infection, vascular or neurological complications, Harlequin syndrome and patient immobility (18). In almost all cases, LV dysfunction is the leading cause for LCOS, making a tailored MCS for LV dysfunction an attractive alternative to ECLS therapy. Furthermore, st-MCS with the Impella pump reduces the need for postoperative blood transfusion and vascular complications, such as limb ischemia compared to VA-ECMO (19, 20). Accordingly, in our cohort no vascular complications occurred.

Length of Impella support strongly varies between different studies, mainly due to heterogeneity of their cohorts (6, 21). In our cohort mean duration of Impella support was 8 ± 5 days with no device malfunctions or device related complications. Four patients received Levosimendan prior to Impella weaning. Meta-analyses indicate that administration of Levosimendan in patients with VA-ECMO has a beneficial effect on weaning success as well as on survival (22, 23). A similar benefit in patients with Impella support remains has yet to be determined. Currently there are no available data to guide the choice of left- vs. right-sided axillary access for Impella implantation. Studies showed that the choice of access side did not affect outcomes or safety, but a shorter delivery time for right sided implantation (24, 25). In our cohort, in 10/11 cases a right sided access was chosen. The most common reason was operator preference and operating room set-up. In three patients with CABG as concomitant procedure, the left internal mammary artery was used as a graft to the left anterior descending. In those cases, as well as in patients with permanent pacemaker the right sided access is preferred.

In st-MCS therapy, timing is an important determinant for a good outcome. Early initiation of Impella support is associated with improved in-hospital and 1-year outcomes, whereas late implantation as a bailout strategy is associated with a higher mortality rate (26, 27). However, in the majority of cardiac operations, st-MCS is used as a rescue therapy, rather than a planned intervention. Few studies and case reports on prophylactic use of the Impella device in high-risk surgery, mainly coronary artery bypass grafting, have already shown encouraging results in prevention of PCCS (28–31). Furthermore, Impella protected PCI is a strategy that results in significantly improved LVEF and survival (32–34). Our results demonstrate the prophylactic Impella support to be a potentially safe and beneficial approach in the management of high-risk mitral valve surgery.

Moreover, this approach also allows for minimal invasive MV surgery, where low LVEF and pulmonary hypertension were previously considered as exclusion criteria for the non-sternotomy approach (10, 35, 36). When planned preoperatively, arterial cannulation for CPB can even be achieved through the right axillary artery via a vascular graft, and venous cannulation can be performed either openly or percutaneously through the groin. It is also possible to gain access to the artery by the Impella and the CPB via a y-graft sutured to the graft anastomosed to the axillary artery. In the case of axillary cannulation without y-Graft, the bypass is gradually weaned and then switched to the Impella device. During this phase, the patient is not supported by CPB or st-MCS but must be bridged with inotropic support. Therefore, in minimal invasive MV surgery we prefer the y-technique or a separate cannulation for the CPB via the groin to prevent a “no-support period”.

The concept of preemptive Impella implantation allows patients with complex structural heart disease to be significantly recruited for a surgical therapy.

### Study limitations

The present study is an observation of clinical parameters, without randomization to different therapies. It is therefore limited by its retrospective and non-randomized nature. Therefore, despite the excellent results, all conclusions are hypothesis-generating and cannot prove superiority of Impella support in high-risk MV surgery over other approaches. Another limitation was the small sample size due to the unique patient cohort. However, to our knowledge this is the first study evaluating preventive Impella implantation in valve surgery. A larger sample size and longer follow-up are necessary to validate the prophylactic use of Impella support in such a patient cohort.

## Conclusion

A combined strategy of surgical treatment of MR and preemptive Impella implantation appears to be a feasible and safe concept. This approach represents an evolution of the concept of “protected cardiac surgery” and may allow for profound treatment of patients with severe MR who would otherwise not be candidates for surgery. The Impella pump therefore provides a tailored mechanical support for the leading cause of heart failure, the insufficient LV, with excellent hemodynamic support to prevent PCCS. This approach might offer an alternative and comprehensive treatment option for patients with MR and severe HFrEF who are not eligible for TEER or in need of concomitant surgery.

## Data Availability

The raw data supporting the conclusions of this article will be made available by the authors, without undue reservation.
